# First molecular detection of *Mycoplasma agassizii* in captive tortoises in Portugal

**DOI:** 10.3389/fvets.2025.1652362

**Published:** 2025-08-25

**Authors:** Manuel Louro, Rui Patrício, André Pereira, Andreia Valença, Margarida Alves

**Affiliations:** ^1^Faculty of Veterinary Medicine, Lusófona University-Lisbon University Centre, Lisbon, Portugal; ^2^Research in Veterinary Medicine (I-MVET), Faculty of Veterinary Medicine, Lusófona University-Lisbon University Centre, Lisbon, Portugal; ^3^All Pets – Clínica Veterinária de Tires, São Domingos de Rana, Portugal; ^4^Superior School of Health, Protection and Animal Welfare, Polytechnic Institute of Lusophony, Lisbon, Portugal; ^5^Animal and Veterinary Research Center (CECAV), Faculty of Veterinary Medicine, Lusófona University - Lisbon University Centre, Lisbon, Portugal; ^6^Global Health and Tropical Medicine (GHTM), Associate Laboratory in Translation and Innovation Towards Global Health (LA-REAL), Instituto de Higiene e Medicina Tropical (IHMT), Universidade NOVA De Lisboa (UNL), Lisbon, Portugal

**Keywords:** captive tortoises, chelonian health, molecular epidemiology, *Mycoplasma agassizii*, polymerase chain reaction, subclinical infection

## Abstract

**Introduction:**

*Mycoplasma agassizii* is a well-recognized etiologic agent of upper respiratory tract disease in tortoises. Although frequently reported in both captive and wild populations across Europe, its occurrence in Portugal had not been previously documented. This study aimed to investigate the presence of *M. agassizii* in apparently healthy captive tortoises in mainland Portugal and to evaluate potential host- and management-related factors associated with infection.

**Methods:**

Oral swabs were collected from 84 tortoises of 13 species across 3 geographic regions. DNA extraction success and sample integrity were confirmed by partial amplification of the tortoise mitochondrial 12S rRNA gene in 92.9% of cases (78/84), which were then screened for *M. agassizii* using a species-specific PCR targeting the 16S rRNA gene.

**Results and discussion:**

The pathogen DNA was detected in 66.7% (52/78) of individuals. Phylogenetic analysis confirmed species identification, with all sequences forming a strongly supported monophyletic cluster together with *M. agassizii* reference sequences. A significant association was observed between tortoise genus and *M. agassizii* infection (*p* = 0.021), with *Chelonoidis* exhibiting a significantly lower infection frequency than Testudo (*p* = 0.029). No statistically significant associations were observed regarding geographic region, housing origin, or group size. These results reveal a high frequency of *M. agassizii* infection in apparently healthy captive tortoises in Portugal, emphasizing its potential for silent transmission in group or mixed-species settings. Our findings support the inclusion of this pathogen in the differential diagnosis of respiratory disease in tortoises, even when clinical signs are absent and underscore the need for routine molecular surveillance and strengthened biosecurity practices to mitigate transmission risks and foster chelonian conservation efforts.

## 1 Introduction

Some *Mycoplasma* species are considered commensals in chelonians, while others can cause severe upper respiratory tract disease (URTD), leading to significant morbidity and mortality in tortoises (1–4). *Mycoplasma agassizii* is the most commonly reported etiologic agent of URTD in both free-ranging and captive tortoises in the United States and Europe (3–9).

*Mycoplasma agassizii* infections may persist in chronic or subclinical forms, with intermittent recurrence of clinical signs such as rhinitis (ranging from mild to severe), nasal and ocular discharges, periocular edema, and conjunctivitis (7–10). Lesions are primarily localized to the nasal cavity, impairing their sense of smell and consequently their ability to forage (8, 11).

Although high pathogen loads are often required to induce disease, subclinically infected tortoises can serve as long-term carriers (8, 12–15). This persistence is likely associated with innate immune responses that reduce but do not eliminate infection, allowing recrudescence under stress conditions (13, 16–18). Persistent infection, combined with pathogen transmission among individuals, contributes to the maintenance and spread of *M. agassizii* within populations (12, 13).

Transmission occurs mainly through direct contact, especially during courtship, mating, or aggressive interactions. Although individuals exhibiting clinical signs are more likely to transmit the pathogen, asymptomatic carriers also play a role in its spread (8, 19, 20). Infection and clinical signs are more frequently reported in captive tortoises, such as those in zoos, rescue centers, breeding facilities, and private collections, likely reflecting increased surveillance and more frequent health assessments. Nevertheless, the close and prolonged contact characteristic of captive settings may also play a significant role in transmission. This has implications for both animal welfare and disease management, with considerable treatment costs (12, 21–25).

Importantly, *M. agassizii* poses a threat to the conservation of wild tortoise populations, particularly those already impacted by anthropogenic pressures (7, 8, 23, 26). Tortoises living near human settlements show higher prevalence compared to those in remote areas with minimal human contact (22, 27), suggesting that the escape or intentional release of captive tortoises may introduce *M. agassizii* and other pathogens, such as herpesvirus, into naïve wild populations (8, 21, 22, 28, 29). Habitat degradation due to agriculture, forestry, mining, urban development, and pollution may further disrupt natural behavior and cause physiological stress, increasing the risk of URTD outbreaks (8, 22, 27, 30). Other stressors include handling, translocations, interactions or injuries involving domestic animals or vehicles, and exposure to waste materials (16, 30–32).

Pathogen surveillance in wildlife is particularly challenging in long-lived hosts and for persistent infections (13). *M. agassizii* is not host-specific and has been detected in several species of free-ranging and captive tortoises (4, 7, 8, 25, 26, 33), raising concerns about interspecies transmission (4, 33). Effective diagnosis and monitoring are therefore crucial, especially in captive populations, to prevent further spread. This is particularly important in the pet trade and conservation programs involving reintroductions, where undetected infections could compromise wild populations. Surveillance of captive tortoises can yield valuable data on the epidemiology of *M. agassizii* and help clarify disease mechanisms, ultimately supporting the prevention and control of URTD in both captive and wild animals (7, 16, 34, 35).

While *M. agassizii* has been reported in both wild and captive tortoises across Europe, no data are currently available on its occurrence in Portugal. This study aimed to investigate the presence of *M. agassizii* in apparently healthy captive tortoises in mainland Portugal and to evaluate potential host- and management-related factors associated with infection.

## 2 Materials and methods

### 2.1 Sample collection

**Figure 1:**
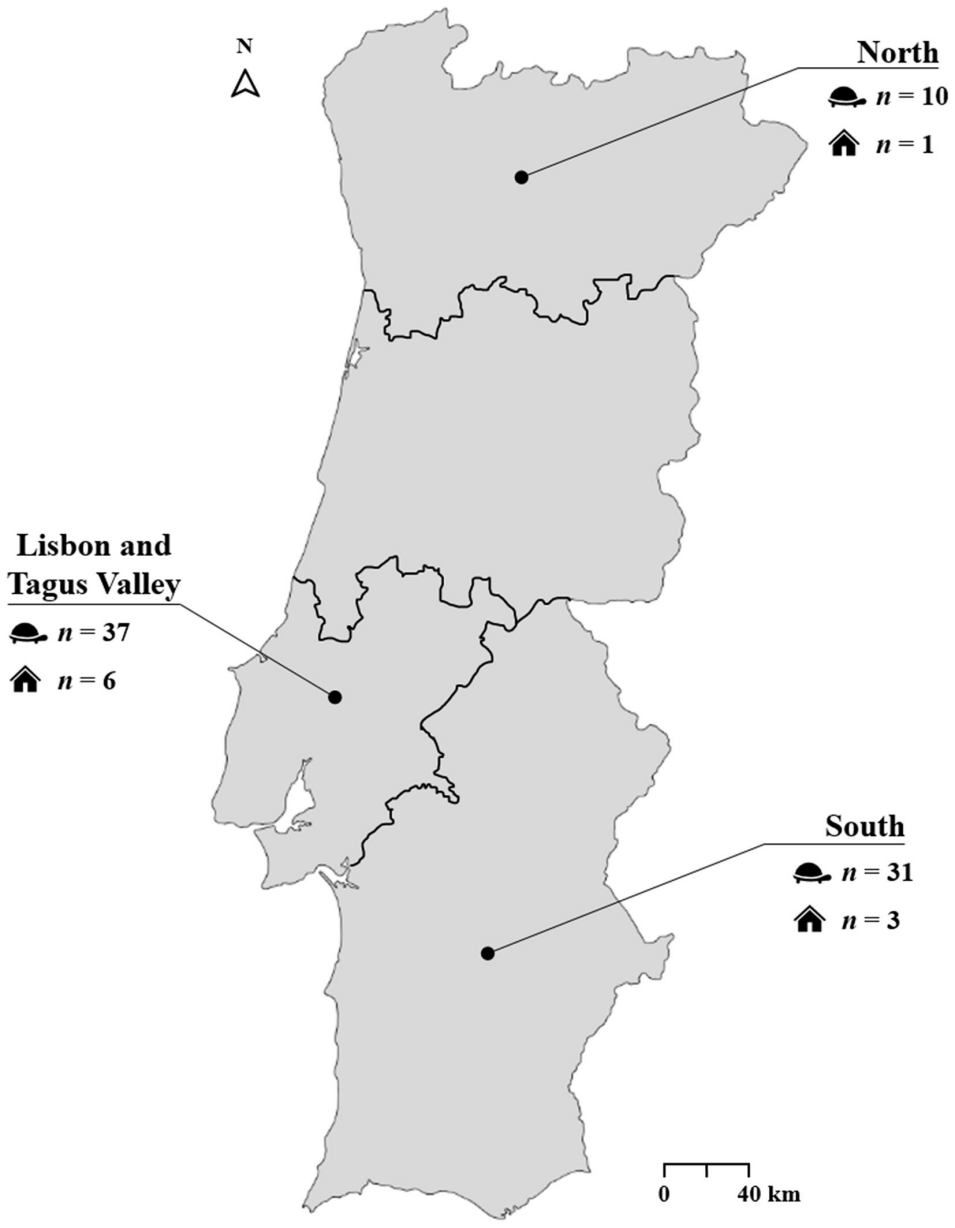
Geographic regions of sample collection.

A cross-sectional survey was conducted between March 2022 and June 2023 in private households (not for commercial or breeding purposes), breeding facilities, and animal parks located in the North, Lisbon and Tagus Valley, and South regions of mainland Portugal (Figure 1). Sterile flocked oral swabs were collected from 84 apparently healthy captive adult tortoises (i.e., no clinical signs compatible with respiratory or other infections were observed during clinical evaluation) of 13 different species, including *Aldabrachelys gigantea* (*n* = 3), *Astrochelys radiata* (*n* = 4), *Centrochelys sulcata* (*n* = 14), *Chelonoidis carbonarius* (*n* = 8), *Geochelone elegans* (*n* = 1), *Indotestudo elongata* (*n* = 2), *Kinixys belliana* (*n* = 1), *Malacochersus tornieri* (*n* = 3), *Stigmochelys pardalis* (*n* = 7), *Testudo graeca* (*n* = 26), *Testudo hermanni* (*n* = 4), *Testudo horsfieldii* (*n* = 3), and *Testudo marginata* (*n* = 2). These animals were distributed across 23 enclosures belonging to 10 different households or facilities. Animals were selected based on availability and accessibility, following a convenience sampling strategy. Immediately after collection, each swab was placed dry (i.e., without transport medium) in a sterile 2 ml microtube. Samples were kept refrigerated (4°C) for a maximum of 24 h after collection and then stored at −80 °C until DNA extraction.

Additionally, each animal caretaker completed a questionnaire designed to gather information on the general health status of the animals, and relevant housing and husbandry practices.

The study was approved by the Ethics and Animal Welfare Committee (CEBEA) of the Faculty of Veterinary Medicine, Lusófona University—Lisbon University Centre (approval number 5/2022). Written informed consent was obtained from all owners or legal detainers prior to sampling.

### 2.2 DNA extraction and chelonian housekeeping gene PCR

DNA was extracted from oral swabs using the Invisorb^®^ Spin Universal Kit (Invitek Molecular, Berlin, Germany), according to the manufacturer's instructions.

Tortoise nucleic acid was detected using primers targeting a conserved region of the mitochondrial 12S rRNA gene (Table 1), as previously described (36). PCR reactions were performed in a total volume of 25 μl, containing 12.5 μl of NZYTaq II 2x Green Master Mix (NZYTech^®^, Lisbon, Portugal), 15 pmol of each primer, 5 μl of total DNA, and sterile ultrapure water to complete the final volume. Non-template controls (NTC) containing water instead of DNA were included in each run. Amplification was carried out on a T100™ Thermal Cycler (Bio-Rad, Hercules, CA, USA) with the following conditions: initial denaturation at 94°C for 5 min; 43 cycles of 94°C for 30 s, 52°C for 45 s, and 72°C for 1 min; followed by a final extension at 72°C for 6 min. PCR products were visualized by electrophoresis on 1.5% (w/v) agarose gels under UV illumination.

**Table 1:** Primers used to amplify tortoise and *Mycoplasma agassizii* DNA.

**Target**	**Primer sequences (5′-3′)**	**Amplicon size**	**References**
mt 12S rRNA gene	Fw: AAAAAGCTTCAAACTGGGATTAGATACCCCACTAT	386 bp	(36)
	Rev: TGACTGCAGAGGGTGACGGGCGGTGTGT		
16S rRNA gene	Fw: CCTATATTATGACGGTACTG	576 bp	(5)
	Rev: TGCACCATCTGTCACTCTGTTAACCTC		

### 2.3 Molecular detection of *M. agassizii*

Only DNA samples in which amplification of the 12S rRNA gene fragment was successfully achieved were screened for *M. agassizii* by PCR targeting the 16S rRNA gene, using species-specific primers previously described by Brown et al. (5) (Table 1).

Reactions were performed in a final volume of 25 μl, containing 12.5 μl of NZYTaq II 2x Green Master Mix (NZYTech^®^), 10 pmol of each primer, 5 μl of DNA, and sterile ultrapure water to complete the final volume. NTC and positive controls (DNA from a confirmed *M. agassizii* sample) were included in each run. Amplification was carried out on a T100™ Thermal Cycler (Bio-Rad) using the following conditions: initial denaturation at 94°C for 5 min; 50 cycles at 94°C for 45 s, 55°C for 1 min, and 72°C for 45 s; followed by a final extension at 72°C for 10 min. Amplicons were visualized by electrophoresis on 1.5% (w/v) agarose gels under UV illumination.

All PCR products with amplicons of the expected size were purified using the Jetquick PCR Product Purification Spin Kit (Genomed GmbH, Bad Oeynhausen, Germany), following the manufacturer's instructions. Purified products were submitted for Sanger sequencing at STABVida^®^ (Caparica, Portugal). Sequence data were analyzed using the FinchTV software (Geospiza^®^), and nucleotide sequence similarity searches were performed with the BLASTn tool (http://blast.ncbi.nlm.nih.gov/Blast.cgi) (37). The nucleotide sequences obtained during this study were deposited in the DDBJ/ENA/GenBank under the accession numbers LC878613-LC878662.

### 2.4 Phylogenetic inference

Multiple sequence alignments were generated using the G-INS-i iterative refinement method implemented in MAFFT v7 (38). The resulting alignments were treated with Gblocks (39), via SeaView v5.0.5, using default parameters. Phylogenetic reconstruction was performed using the maximum likelihood method implemented in IQ-TREE v1.6.12, with the best-fit substitution model selected according to the Bayesian Information Criterion. Branch support was assessed using the bootstrap method with 1,000 replicates, with values ≥75% considered indicative of strong topological support. Trees were visualized and edited for display using iTOL v6 (40). A list of all *Mycoplasma* spp. sequences included in the phylogenetic analysis, along with associated metadata (i.e., strain name or molecular ID, host, country of origin, GenBank accession number, and type strain status), is provided in Supplementary Table 1.

### 2.5 Statistical analysis

Data were compiled in Microsoft Excel v365 and analyzed using IBM SPSS Statistics v25. Exploratory and descriptive analyses were conducted to characterize the dataset. Relative frequencies of *M. agassizii*-positive cases were calculated, and 95% confidence intervals (CI) were obtained using Wilson's method via the OpenEpi online tool (41).

Associations between categorical variables (i.e., tortoise species, geographic region, origin, and housing type) and *M. agassizii* infection status were explored using the Chi-square (χ^2^) test, Fisher's exact test (for 2 × 2 tables), or the Fisher–Freeman–Halton exact test (for larger contingency tables), as appropriate. Adjusted standardized residuals (ASR) were determined to identify cells with significant contributions to the overall test result, with ASR values exceeding ±1.96 considered statistically significant (α = 0.05). Pairwise comparisons were conducted between categories of variables that showed significant overall differences.

The Mann–Whitney U test was used to evaluate differences in the number of animals per enclosure between groups defined by *M. agassizii* infection status. A *p*-value < 0.05 was considered statistically significant for all analyses.

## 3 Results

Partial amplification of the mitochondrial 12S rRNA gene, employed as a host-specific internal control, was successful in 92.9% (78/84) of the sampled tortoises. These 78 samples were considered suitable for further molecular screening of *M. agassizii*.

*Mycoplasma agassizii* 16S rRNA gene fragment was detected in 66.7% (52/78) of the screened individuals (Table 2). BLASTn analysis of the obtained sequences showed 100% identity and 99% query cover with *M. agassizii* reference sequences available in public databases (e.g., GenBank accession no. KY212532).

**Table 2:** Molecular frequency of *Mycoplasma agassizii* infection in captive chelonians by species, origin, region, and housing conditions.

**Variables/categories**	**Tested**	**Positive**	**95% CI**	***p*-value/ASR**
Genus/species, *n* (%)				*p* = 0.021
*Aldabrachelys*	3 (3.8)	1 (33.3)	6.2–79.2	−1.2
*A. gigantea*	3 (100)	1 (33.3)	6.2–79.2	
*Astrochelys*	4 (5.1)	4 (100)	51.0–100	1.5
*Astrochelys radiata*	4 (100)	4 (100)	51.0–100	
*Centrochelys*	14 (17.9)	7 (50.0)	26.8–73.2	−1.5
*C. sulcata*	14 (100)	7 (50.0)	26.8–73.2	
*Chelonoidis*	8 (10.3)	2 (25.0)^a^	7.1–59.1	−2.6
*C. carbonarius*	8 (100)	2 (25.0)	7.1–59.1	
*Geochelone*	1 (1.3)	1 (100)	20.7–100	0.7
*G. elegans*	1 (100)	1 (100)	20.7–100	
*Indotestudo*	2 (2.6)	2 (100)	34.2–100	1.0
*I. elongata*	2 (100)	2 (100)	34.2–100	
*Kinixys*	1 (1.3)	0 (0.0)	0.0–79.3	−1.4
*K. belliana*	1 (100)	0 (0.0)	0.0–79.3	
*Malacochersus*	3 (3.8)	3 (100)	43.9–100	1.2
*M. tornieri*	3 (100)	3 (100)	43.9–100	
*Stimochelys*	7 (9.0)	6 (85.7)	48.7–97.4	1.1
*S. pardalis*	7 (100)	6 (85.7)	48.7–97.4	
*Testudo*	35 (44.9)	26 (74.3)^a^	57.9–85.9	1.3
*T. graeca*	26 (74.3)	19 (73.1)	53.9–86.3	
*T. hermanni*	4 (11.4)	3 (75.0)	30.1–95.4	
*T. horsfieldii*	3 (8.6)	3 (100)	43.9–100	
*T. marginata*	2 (5.7)	1 (50.0)	9.5–90.6	
Geographic region, *n* (%)				*p* = 0.145
North	10 (12.8)	4 (40.0)	16.8–68.7	−1.9
Lisbon and Tagus Valley	37 (47.4)	27 (73.0)	57.0–84.6	1.1
South	31 (39.7)	21 (67.7)	50.1–81.4	0.2
Origin, *n* (%)				*p* = 0.651
Animal park	31 (39.7)	21 (67.7)	50.1–81-4	0.2
Breeder	28 (35.9)	17 (60.7)	42.4–76.4	−0.8
Private owner	19 (24.4)	14 (73.7)	51.2–88.2	0.7
Individual housing, *n* (%)				*p* = 0.342
No	72 (92.3)	47 (65.3)	53.8–75.3	0.9
Yes	6 (7.7)	5 (83.3)	43.7–97.0	−0.9
Enclosure group size, median (IQR)	4.0	4.0 (4.8)		*p* = 0.801
Total	78	52 (66.7)	55.5–76.1	

^a^*p* = 0.029.

ASR, Adjusted standardized residuals; CI: 95%, Confidence interval.

**Figure 2:**
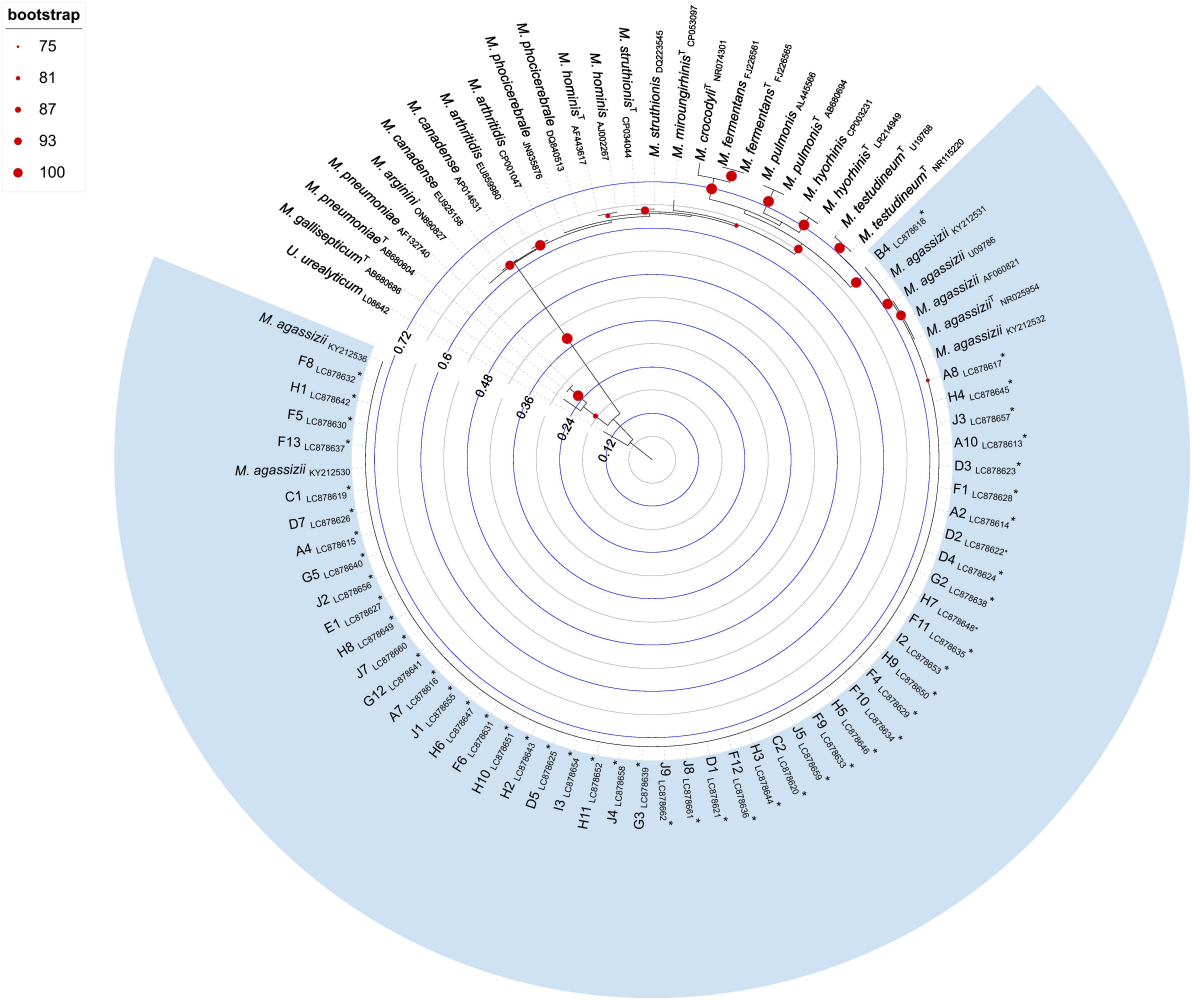
Maximum likelihood phylogenetic tree inferred from *Mycoplasma* spp. 16S rRNA gene sequences (509 bp). Tree reconstruction was performed in IQ-TREE using the TVMe+I+G4 substitution model, selected as the best-fitting model based on the Bayesian Information Criterion. Node support was assessed using 1,000 bootstrap replicates, and values ≥75% are shown at the corresponding nodes. The tree was rooted using a *Ureaplasma urealyticum* sequence (GenBank accession number: L08642). Sequences retrieved from public databases are labeled with species name and GenBank accession number; type strains are indicated with a superscript “T” following the species name. Sequences obtained in this study are marked with an asterisk and include the tortoise identifier and GenBank accession numbers LC878613–LC878662. Branch lengths are proportional to the number of nucleotide substitutions per site, as indicated by the internal scale bar.

Phylogenetic analysis revealed that all 16S rRNA sequences obtained in this study segregated into a well-supported monophyletic cluster, exclusively composed of *M. agassizii* reference sequences (Figure 2).

Infection frequencies across species ranged from 0.0% in *K. belliana* (*n* = 1) to 100% in several species, including *A. radiata* (*n* = 4), *I. elongata* (*n* = 2), *M. tornieri* (*n* = 3), *G. elegans* (*n* = 1), and *T. horsfieldii* (*n* = 3). Although these species had small sample sizes, *T. graeca* had the highest number of individuals testing positive for *M. agassizii* DNA (19/26).

Among genera with at least three sampled individuals, *Testudo* showed the highest detection frequency (74.3%; 26/35). A statistically significant association was observed between tortoise genus and the presence of *M. agassizii* DNA (*p* = 0.021; Table 2). Pairwise comparison showed that *Chelonoidis* had a significantly (*p* = 0.029) lower frequency (25.0%, 2/8; ASR = −2.6,) of *M. agassizii* infection compared to other genera, particularly in contrast to *Testudo* (74.3%, 26/35).

Positive cases were detected across all sampled regions in mainland Portugal. The Lisbon and Tagus Valley region recorded the highest number of infected tortoises (73.0%, 27/37), followed by the South (67.7%, 21/31) and the North (40.0%, 4/10). However, these regional differences were not statistically significant (*p* = 0.145).

*Mycoplasma agassizii* DNA was detected in tortoises from all housing origins: 73.7% (14/19) of private households, 67.7% (21/31) of animal parks, and 60.7% (17/28) of breeders. No statistically significant association was found between housing origin and infection status (*p* = 0.651).

Regarding housing conditions, no association was found between *M. agassizii* DNA detection and enclosure group size or individual housing. The median number of animals per enclosure did not differ significantly between infected and uninfected tortoises (*p* = 0.801), nor was any association found with being housed individually (*p* = 0.342).

## 4 Discussion

While *M. agassizii* has been previously reported in both wild and captive tortoises across Europe, this is the first comprehensive study to assess its presence in captive tortoises kept under diverse housing origins and conditions in mainland Portugal. The high detection frequency (66.7%; 52/78) aligns with *M. agassizii* transmission dynamics, which rely on prolonged direct contact (typically >24–48 h) for successful spread (12). Such conditions are frequently met in captive settings due to co-housing and group management practices (12, 21–25). However, this percentage is higher than those reported in both free-ranging and captive tortoises in other European countries, where prevalence ranged from 0 to 42% (4, 7, 25, 42, 43). Notably, unlike previous studies, none of the animals tested in this study exhibited clinical signs of URTD, suggesting subclinical or chronic infections. By acting as reservoirs, these individuals can contribute to pathogen persistence and spread, complicating sanitary control, especially given the lower pathogen loads often observed in subclinical cases (8, 12–15). This highlights the importance of molecular screening, even in the absence of clinical signs, particularly during quarantine, relocation, or introduction into new enclosures.

Although quarantine periods of 12–18 months are recommended (8, 44), they are often impractical or omitted altogether, especially in illicit trade contexts. Furthermore, subclinical infections may remain undetected throughout quarantine. Therefore, rapid molecular screening at intake, using oral swabs (a non-invasive method compatible with the epithelial tropism of mycoplasmas) offers a practical approach for *M. agassizii* routine testing.

Most animals in this study were not housed individually, increasing the risk of horizontal transmission. This raises the possibility that some individuals may have become infected only after being introduced in mixed enclosures, thereby underlining the risk of within-collection transmission. Longitudinal follow-up of the six enclosures where both positive and negative individuals were detected could provide further insight into transmission dynamics and infection timelines.

Interestingly, in this study, tortoises of the genus *Testudo* exhibited a significantly higher infection frequency than those of the genus *Chelonoidis*. Although tortoises are not autochthonous to Portugal, *Testudo* species are native to neighboring Mediterranean countries, such as Spain, France, Italy, and Greece (45). The decline of wild *Testudo* populations is well documented, with IUCN conservation statuses ranging from Near Threatened to Critically Endangered (3, 43). These populations face numerous threats including habitat loss and fragmentation, urban development, predation, delayed maturity, low fecundity, illegal pet trade and movement of exotic animals, competition with exotic tortoise species, and infectious diseases such as URTD (7, 8, 23, 46, 47).

The international pet trade exacerbates conservation challenges by enabling long-distance movement of infected individuals with limited or no sanitary oversight (35, 48). These flows of individuals result in open routes for the expansion of emerging infectious pathogens, such as *M. agassizii*. Given its high prevalence in captive animals, the introduction of infected individuals into the wild, whether intentional or accidental, could have devastating consequences for naïve wild populations. Studies have shown higher *M. agassizii* prevalence in tortoises associated with proximity to human settlements, likely reflecting contacts with captive or released individuals (22, 27). Ballouard et al. (42) reported high *M. agassizii* infection rates in captive and vagrant exotic tortoises co-occurring with native *T. h. hermanni* in urban or peri-urban areas in southeastern France. These findings raise concerns about the uncontrolled introduction of exotic pet tortoises and the potential transmission from captive to free-ranging tortoises. Conversely, the illegally harvesting of wild tortoises can also introduce *M. agassizii* into captive settings, thus perpetuating the transmission cycle.

Cross-species transmission of *M. agassizii* has been documented (4, 33), and many positive animals in this study were housed in mixed-species enclosures, supporting the likelihood of interspecies transmission. The fact that *M. agassizii* was detected in 12 different tortoise species, including *C. sulcata, C. carbonarius, I. elongata*, and *M. tornieri*, confirms that this pathogen is not species-specific. This contrasts with recent findings from Galosi et al. (7), who did not detect the pathogen in these species in Italy. Further research on both mixed-species and species-specific enclosures may provide new insights into cross-species transmission dynamics.

Several studies have reported URTD-like syndromes in mycoplasma-positive turtles of the family Emydidae (2, 25, 49–52). Although *M. agassizii* primarily affects tortoises, its full host range and transmission dynamics remain incompletely understood. There is some indication of *Mycoplasma* spp. being detected in freshwater turtles, including unpublished data suggesting PCR detection of *M. agassizii* in *Trachemys scripta elegans* (8). Further studies are needed to assess the susceptibility of other chelonian species and to evaluate potential risks associated with interspecific contact in shared environments. Given the ecological overlap between tortoises and freshwater turtle populations, investigating the potential for cross-species transmission remains an important area for future research.

Despite the absence of clinical signs in the tortoises sampled for this study, *M. agassizii* is recognized as the primary etiological agent of URTD in tortoises (3–9). This infection, even when subclinical, has the potential to progress to overt disease under certain stressors or immunosuppressive conditions (7–10, 13, 16–18). The high frequency observed here reinforces the need to include *M. agassizii* in differential diagnoses of respiratory conditions in tortoises, particularly in captive settings, new acquisitions, or individuals with recent chelonian contact. This study provides valuable epidemiological evidence to inform clinical decision-making and improve biosecurity protocols in both private and institutional collections.

## 5 Conclusion

This study provides the first evidence of widespread *M. agassizii* infection among captive tortoises in mainland Portugal. The pathogen was detected in multiple tortoise species, including species native to the Mediterranean region, yet none exhibited clinical signs at the time of sampling. This highlights the potential role of subclinical carriers in maintaining and spreading the infection, especially in settings with group housing or mixed-species enclosures. The international reptile trade, coupled with insufficient sanitary oversight, remains a driver of pathogen dissemination, posing significant risks to both native and non-native chelonian populations.

Given *M. agassizii*'s established role in URTD and its potential to cause clinical illness under stress or immunosuppression, the results presented here support its inclusion in the differential diagnosis of respiratory disease in tortoises, even in the absence of clinical signs. Furthermore, our results underscore the importance of incorporating molecular diagnostics as a standard practice in routine veterinary care and tortoise collection management. This study offers valuable insights for guiding effective strategies to prevent pathogen transmission and protect vulnerable tortoise populations.

## Data Availability

The datasets presented in this study can be found in online repositories. The names of the repository/repositories and accession number(s) can be found below: https://www.ncbi.nlm.nih.gov/genbank/, LC878613-LC878662.
