# Supplementary material for: First molecular detection of Mycoplasma agassizii in captive tortoises in Portugal
Source: Front Vet Sci. 2025 Aug 25;12:1652362. doi: 10.3389/fvets.2025.1652362 (PMC12416086; doi:10.3389/fvets.2025.1652362)
Supplement: Supplementary file 1 [file Table_1.docx]

Supplementary Material

**Supplementary Table 1.** Metadata associated with *Mycoplasma* spp. sequences included in the phylogenetic analysis.

| **Species^a^** | **Strain/Molecular ID** | **Host (common name/species)** | **Country** | **GenBank Accession Number** |
| --- | --- | --- | --- | --- |
| *M. agassizii* | K120 | n.a. | n.a. | AF060821 |
| *M. agassizii* | n.a. | Desert tortoise/*Gopherus agassizii* | n.a. | U09786 |
| *M. agassizii* | UFGb40 | Texas tortoise/*Gopherus berlandieri* | USA | KY212531 |
| *M. agassizii* | UFIf01 | Forsten’s tortoise/*Indotestudo forstenii* | USA | KY212532 |
| *M. agassizii* | UFPp01 | Flat tailed tortoise/*Pyxis planicauda* | USA | KY212530 |
| *M. agassizii* | UFCs01 | African spurred tortoise/*Centrochelys sulcata* | USA | KY212536 |
| *M. agassizii* | ATCC 700616^T^ | Desert tortoise/*Gopherus agassizii* | USA | NR025954 |
| *M. agassizii* | A2* | Leopard tortoise/*Stigmochelys pardalis* | Portugal | LC878614 |
| *M. agassizii* | A4* | Greek tortoise/*Testudo graeca* | Portugal | LC878615 |
| *M. agassizii* | A7* | Greek tortoise/*Testudo graeca* | Portugal | LC878616 |
| *M. agassizii* | A8* | Sulcata tortoise/*Centrochelys sulcata* | Portugal | LC878617 |
| *M. agassizii* | A10* | Greek tortoise/*Testudo graeca* | Portugal | LC878613 |
| *M. agassizii* | B4* | Leopard tortoise/*Stigmochelys pardalis* | Portugal | LC878618 |
| *M. agassizii* | C1* | Sulcata tortoise/*Centrochelys sulcata* | Portugal | LC878619 |
| *M. agassizii* | C2* | Sulcata tortoise/*Centrochelys sulcata* | Portugal | LC878620 |
| *M. agassizii* | D1* | Radiated tortoise/*Astrochelys radiata* | Portugal | LC878621 |
| *M. agassizii* | D2* | Radiated tortoise/*Astrochelys radiata* | Portugal | LC878622 |
| *M. agassizii* | D3* | Leopard tortoise/*Stigmochelys pardalis* | Portugal | LC878623 |
| *M. agassizii* | D4* | Leopard tortoise/*Stigmochelys pardalis* | Portugal | LC878624 |
| *M. agassizii* | D5* | Leopard tortoise/*Stigmochelys pardalis* | Portugal | LC878625 |
| *M. agassizii* | D7* | Leopard tortoise/*Stigmochelys pardalis* | Portugal | LC878626 |
| *M. agassizii* | E1* | Aldabra giant tortoise/*Aldabrachelys gigantea* | Portugal | LC878627 |
| *M. agassizii* | F1* | Marginated tortoise/*Testudo marginata* | Portugal | LC878628 |
| *M. agassizii* | F4* | Hermann’s tortoise/*Testudo hermanni* | Portugal | LC878629 |
| *M. agassizii* | F5* | Hermann’s tortoise/*Testudo hermanni* | Portugal | LC878630 |
| *M. agassizii* | F6* | Hermann’s tortoise/*Testudo hermanni* | Portugal | LC878631 |
| *M. agassizii* | F8* | Greek tortoise/*Testudo graeca* | Portugal | LC878632 |
| *M. agassizii* | F9* | Greek tortoise/*Testudo graeca* | Portugal | LC878633 |
| *M. agassizii* | F10* | Greek tortoise/*Testudo graeca* | Portugal | LC878634 |
| *M. agassizii* | F11* | Russian tortoise/*Testudo horsfieldii* | Portugal | LC878635 |
| *M. agassizii* | F12* | Russian tortoise/*Testudo horsfieldii* | Portugal | LC878636 |
| *M. agassizii* | F13* | Russian tortoise/*Testudo horsfieldii* | Portugal | LC878637 |
| *M. agassizii* | G2* | Elongated tortoise/*Indotestudo elongata* | Portugal | LC878638 |
| *M. agassizii* | G3* | Elongated tortoise/*Indotestudo elongata* | Portugal | LC878639 |
| *M. agassizii* | G5* | Red-footed tortoise/*Chelonoidis carbonarius* | Portugal | LC878640 |
| *M. agassizii* | G12* | Red-footed tortoise/*Chelonoidis carbonarius* | Portugal | LC878641 |
| *M. agassizii* | H1* | Greek tortoise/*Testudo graeca* | Portugal | LC878642 |
| *M. agassizii* | H2* | Greek tortoise/*Testudo graeca* | Portugal | LC878643 |
| *M. agassizii* | H3* | Greek tortoise/*Testudo graeca* | Portugal | LC878644 |
| *M. agassizii* | H4* | Greek tortoise/*Testudo graeca* | Portugal | LC878645 |
| *M. agassizii* | H5* | Sulcata tortoise/*Centrochelys sulcata* | Portugal | LC878646 |
| *M. agassizii* | H6* | Sulcata tortoise/*Centrochelys sulcata* | Portugal | LC878647 |
| *M. agassizii* | H7* | Sulcata tortoise/*Centrochelys sulcata* | Portugal | LC878648 |
| *M. agassizii* | H8* | Sulcata tortoise/*Centrochelys sulcata* | Portugal | LC878649 |
| *M. agassizii* | H9* | Pancake tortoise/*Malacochersus tornieri* | Portugal | LC878650 |
| *M. agassizii* | H10* | Pancake tortoise/*Malacochersus tornieri* | Portugal | LC878651 |
| *M. agassizii* | H11* | Pancake tortoise/*Malacochersus tornieri* | Portugal | LC878652 |
| *M. agassizii* | I2* | Radiated tortoise/*Astrochelys radiata* | Portugal | LC878653 |
| *M. agassizii* | I3* | Radiated tortoise/*Astrochelys radiata* | Portugal | LC878654 |
| *M. agassizii* | J1* | Greek tortoise/*Testudo graeca* | Portugal | LC878655 |
| *M. agassizii* | J2* | Greek tortoise/*Testudo graeca* | Portugal | LC878656 |
| *M. agassizii* | J3* | Greek tortoise/*Testudo graeca* | Portugal | LC878657 |
| *M. agassizii* | J4* | Greek tortoise/*Testudo graeca* | Portugal | LC878658 |
| *M. agassizii* | J5* | Greek tortoise/*Testudo graeca* | Portugal | LC878659 |
| *M. agassizii* | J7* | Greek tortoise/*Testudo graeca* | Portugal | LC878660 |
| *M. agassizii* | J8* | Greek tortoise/*Testudo graeca* | Portugal | LC878661 |
| *M. agassizii* | J9* | Greek tortoise/*Testudo graeca* | Portugal | LC878662 |
| *M. arginini* | 12686 | Bovine/n.a. | USA | ON890827 |
| *M. arthritidis* | 91021 | n.a. | n.a. | EU859980 |
| *M. arthritidis* | 158L3-1 | Rat/*Rattus* spp. | n.a. | CP001047 |
| *M. canadense* | 466 | n.a. | n.a. | EU925158 |
| *M. canadense* | HAZ360_1 | Bovine/n.a. | Japan | AP014631 |
| *M. crocodyli* | MP145^T^ | Crocodile/*Crocodylus niloticus* | Zimbabwe | NR074301 |
| *M. fermentans* | PG-18^T^ | Human/*Homo sapiens* | USA | FJ226561 |
| *M. fermentans* | M39 | Human/*Homo sapiens* | n.a. | FJ226565 |
| *M. gallisepticum* | NBRC 14855^T^ | Chicken/n.a. | UK | AB680686 |
| *M. hominis* | ATCC 23114^T^ | Human/*Homo sapiens* | n.a. | AF443617 |
| *M. hominis* | SC4 | Human/*Homo sapiens* | n.a. | AJ002267 |
| *M. hyorhinis* | GDL-1 | n.a. | n.a. | CP003231 |
| *M. hyorhinis* | NCTC10121^T^ | n.a. | n.a. | LR214949 |
| *M. miroungirhinis* | ES2806-NAS^T^ | Northern elephant seal/*Mirounga angustirostris* | USA | CP053097 |
| *M. phocicerebrale* | Cheryl | n.a. | n.a. | JN935876 |
| *M. phocicerebrale* | CSL 5195S2 | n.a. | n.a. | DQ840513 |
| *M. pneumoniae* | NBRC 14401^T^ | Human/*Homo sapiens* | USA | AB680604 |
| *M. pneumoniae* | ATCC 15531 | Human/*Homo sapiens* | USA | AF132740 |
| *M. pulmonis* | UAB CTIP | Rat/*Rattus* spp. | USA | AL445566 |
| *M. pulmonis* | NBRC 14896^T^ | Rat/*Rattus* spp. | USA | AB680694 |
| *M. struthionis* | 237IA^T^ | Ostrich/*Struthio camelus* | Namibia | CP034044 |
| *M. struthionis* | Ms01 | Ostrich/*Struthio camelus* | South Africa | DQ223545 |
| *M. testudineum* | H3110^T^ | Desert tortoise/*Gopherus agassizii* | n.a. | U19768 |
| *M. testudineum* | BH29^T^ | Desert tortoise/*Gopherus agassizii* | USA | NR115220 |

* Sequence generated in the present study; ^T^ Type strain; ^a^ species designation according to the original depositors; n.a. = not available.

Note: The table presents metadata corresponding to *Mycoplasma* spp. 16S rRNA sequences used in the phylogenetic analysis. For each entry, the species name, strain or molecular identifier, host (common and scientific names), country of origin, and GenBank accession number are indicated when available. All metadata were retrieved from the NCBI Nucleotide database or, where necessary, supplemented with information from BacDive.
